# Gestión informativa de la infodemia en medios digitales: experiencia de las agencias de noticias

**DOI:** 10.26633/RPSP.2021.25

**Published:** 2021-05-12

**Authors:** María Victoria González Clavero, Grettel Rodríguez Bazán

**Affiliations:** 1 Facultad de Humanidades, Universidad Central “Marta Abreu” de Las Villas Santa ClaraVilla Clara Cuba Facultad de Humanidades, Universidad Central “Marta Abreu” de Las Villas, Santa Clara, Villa Clara, Cuba.

**Keywords:** Comunicación en salud, periodismo, pandemias, Health communication, journalism, pandemics, Comunicação em saúde, jornalismo, pandemias

## Abstract

En la presente comunicación se propone una guía para gestionar la infodemia en medios digitales, a partir de las experiencias de agencias de noticias en momentos de confluencia de la creciente producción periodística y el enfrentamiento a la epidemia de COVID-19. Se realizó una revisión bibliográfica documental de artículos científicos y documentos de organismos internacionales sobre infodemia, infodemiología y gestión de la información periodística en casos de desastres y emergencias, así como el análisis de las secciones de salud y de verificación en los sitios de Internet y redes sociales de las agencias Reuters, AFP y EFE. La experiencia acumulada devela no solo la utilidad sino también la necesidad de que los medios de comunicación recurran al uso del análisis de los datos, el periodismo de servicio, la anticipación y prevención de información falsa, la colaboración, la narrativa a través de diferentes medios de comunicación y la alfabetización mediática tanto de los consumidores de la información como de los que la producen. Se propone un plan de 10 acciones (agrupadas en tres etapas: recogida de información, selección de la información periodística y presentación de la información) que pueden guiar a los medios informativos digitales en el combate contra la desinformación en temas de salud.

## Abstract (en)

This article offers guidance on managing the infodemic in digital media, based on the experiences of news agencies at a time when an increase in journalistic production is coinciding with the response to the COVID-19 pandemic. A documentary review of the literature (scientific articles and documents from international agencies) on infodemics, infodemiology, and management of journalistic information during disasters and emergencies was carried out, as well as an analysis of the health and fact-checking sections on the websites and social networks of the Reuters, AFP and EFE news agencies. The accumulated experience reveals that it is not only useful but necessary for the media to make use of data analysis, service journalism, anticipation and prevention of false information, collaboration, narratives across different media, and media literacy both in consumers and producers of information. This article proposes a plan consisting of 10 actions (grouped into three stages: information collection, selection of journalistic information, and presentation of information) that can help digital media combat misinformation about health issues.

## RESUMO

Nesta comunicação é proposto um guia para o controle da infodemia nas mídias digitais a partir da experiência adquirida pelas agências de notícias com a confluência entre a crescente produção jornalística e o enfrentamento à pandemia de COVID-19. Foi realizada uma revisão documental de artigos científicos e documentos de organismos internacionais sobre infodemia, infodemiologia e gestão da informação jornalística em desastres e emergências, bem como a análise das seções de saúde e checagem de fatos de sites e redes sociais das agências Reuters, AFP e EFE. A experiência acumulada demonstra não só a utilidade, mas também a necessidade de a mídia recorrer a análise de dados, serviço jornalístico, mecanismos para antecipar e prevenir fake news, colaboração, narrativa por diferentes mídias e educação midiática tanto dos consumidores da informação como dos que a produzem. É proposto um plano de 10 ações em três etapas – coleta de informação, seleção da informação jornalística e apresentação da informação – para servir de guia às mídias informativas digitais no combate à desinformação em saúde.

La sobreabundancia de información inexacta, engañosa y francamente falsa se ha convertido en una pandemia que afecta al mundo desde hace ya varios años. Los cambios de roles de las audiencias, el acceso masivo a Internet y la figura del productor-consumidor de información o *prosumidor* trastocan los códigos de la comunicación e imponen retos al periodismo. Por otra parte, la politización de las redes sociales y su manejo con fines de lucro económico —y de otras índoles— las convierten en escenarios propicios para la difusión de contenidos engañosos y falsos. De hecho, se afirma que el panorama creado por el exceso de información dudosa empeora más la ya deteriorada situación informativa, ya que las noticias falsas se propagan en las redes sociales mucho más rápidamente que las verdaderas (1).

La crisis desatada por la epidemia causada por el SARS-CoV-2 tiene diversas aristas, ya que el desconcierto y la consternación por su arista sanitaria genera un terreno fértil para la avalancha informativa que obstaculiza el poder discernir lo auténtico de lo falso. Esto ha generado la aparición de nuevas disciplinas, como la infovigilancia (análisis y seguimiento de la información publicada relacionada con la salud) y la infodemiología (la ciencia de la gestión de infodemias). Desde hace algunos años, han surgido proyectos colaborativos dirigidos a elevar la calidad y la veracidad de la información que se publica. Desde el año 2015, la organización International Fact-Checking Network (IFCN) monitorea y promueve el análisis generalizado de la información, independientemente de su procedencia. Otros proyectos de verificación de noticias han ganado reconocimiento y prestigio, entre ellos Latam-Chequea, enfocado en los países latinoamericanos, y Maldita.es y Newtral, ambos radicados en España. En un estudio publicado en 2020 se reconoce que alrededor de la tercera parte de las informaciones falsas que se propagan en las redes sociales tratan sobre salud (2).

Diversos organismos internacionales, como la Organización Mundial de la Salud (OMS), la Organización Panamericana de la Salud (OPS) y la Organización de las Naciones Unidas para la Educación, la Ciencia y la Cultura (UNESCO), sitúan el periodismo en un primer plano en la respuesta general a la infodemia (3, 4). Si bien la prensa ha sufrido una pérdida de confianza y la llamada “desinfodemia” (diseminación abundante de información inexacta, engañosa y falsa) menoscaba la labor de los medios y los periodistas (5), la verificación de los contenidos ofrece a los periodistas y otros profesionales de la información la oportunidad de restituir su dañada credibilidad (6) mediante una intervención eficaz.

El tratamiento periodístico de temas relacionados con la salud experimenta un considerable auge (7) ante la demanda creciente por parte de los receptores de información (8). Sin embargo, la inmediatez requerida y la excesiva abundancia informativa imponen retos a los periodistas especializados en temas sanitarios: la actualización constante, el desarrollo de habilidades para el manejo de datos científicos y la curación de contenidos (9).

Ante esta problemática, las agencias de noticias de alcance global —como Reuters, AFP y EFE, entre otras— se implican cada vez más en la verificación de las noticias sobre salud y ofrecen sus contenidos gratuitamente al público general y especializado.

En la presente comunicación se presenta una propuesta de acciones por etapas para gestionar la infodemia en medios digitales, a partir de las experiencias de agencias de noticias en momentos de confluencia de la creciente producción periodística y el enfrentamiento a la pandemia de COVID-19.

## INSUMOS PARA EL ANÁLISIS DE LA SITUACIÓN

Se realizó una revisión bibliográfica documental de artículos científicos y documentos de la OMS, la OPS y la UNESCO sobre infodemia, infodemiología, y gestión de la información periodística en casos de desastres y emergencias.

Para conocer sobre la respuesta dada por las agencias de noticias a la infodemia, se analizaron las secciones de salud y de verificación —específicamente en lo concerniente a la COVID-19— en los sitios de Internet y redes sociales de las agencias Reuters, AFP y EFE, consideradas por muchos como modelos dentro del sistema de medios periodísticos. Además, se consultaron documentos emitidos por la agencia Reuters sobre la validación de contenidos (alfabetización mediática de los consumidores de información) y entrevistas sobre el tema a la responsable del sitio EFE Verifica. Las experiencias positivas de las agencias de noticias pueden contribuir a la gestión de la infodemia en medios digitales en general.

En este artículo se presenta un plan de acciones que puede ayudar a los medios informativos digitales a combatir la desinformación en temas de salud.

## CLAVES PARA COMBATIR LA INFODEMIA EN LAS AGENCIAS DE NOTICIAS

Según un análisis realizado por la OMS, “las infodemias, como las epidemias, se pueden controlar. Si la epidemiología de campo implica: a) el seguimiento y la identificación de las amenazas para la salud, b) la investigación de los brotes y c) las acciones de mitigación y control, el manejo de la infodemia se debe basar en: a) el monitoreo y la identificación del problema, b) el análisis de la información y c) medidas de control y mitigación” (10).

Las agencias de noticias son reconocidas seleccionadoras de hechos noticiables y fuentes del sistema de medios de comunicación y, como tales, suelen responder con inmediatez a las exigencias del mercado informativo gracias a su elevada capacidad tecnológica y de innovación.

Durante el 6.° Congreso Mundial de Agencias de Noticias, celebrado en Bulgaria en 2019, se llegó al consenso de dar prioridad al enfrentamiento a las noticias falsas y engañosas, conocidas ya universalmente como *fake news* (11), un componente de la infodemia que afecta a sus clientes.

En las agencias de noticias, la lucha contra la infodemia se debe basar en cuatro pilares: la verificación, la colaboración, el servicio y la alfabetización mediática de los consumidores y los productores de información.

El periodismo de verificación, en la modalidad de comprobación de los hechos contra las noticias falsas, conocida como *fact-checking vs. fake news*, propicia el desmontaje de temas proclives a la manipulación, como la salud. Los sitios en Internet de Reuters, AFP y EFE ofrecen contenidos gratuitos que guían a la opinión pública. Su credibilidad descansa no solo en el prestigio ganado, sino también en herramientas específicas propias —como *Trust Principles* (Reuters) y Reglas de Buenas Prácticas Editoriales y Deontológicas (AFP)— o de organizaciones internacionales externas —como *CrossCheck*, *First Draft*, *Comprova* (AFP), e *IFCN* (AFP y EFE)—, entre otras.

Además, estas agencias colaboran con entidades homólogas, gremios de salud y redes sociales, como Facebook, para la confirmación de contenidos sospechosos y propensos a convertirse en foco de atención o “viralización”. Las agencias AFP y EFE extienden sus páginas de verificaciones a Facebook y Twitter, dos de los canales más frecuentemente empleados para la difusión de noticias falsas.

Los departamentos de verificación de estas agencias incorporan a periodistas experimentados a la comprobación e investigación de hechos, de conjunto con expertos y el uso de ciertas tecnologías de avanzada. En esencia, su estrategia se basa en la curación de contenidos: “filtrado, selección, ordenación y difusión de información proveniente de fuentes diversas” (12).

La detección del llamado ruido informativo se produce a partir del monitoreo de Internet, redes sociales y comunicaciones enviadas al medio. Los criterios de selección establecidos por Reuters son el mérito editorial, el nivel de “viralidad” y el equilibrio entre información y opinión. Por su parte, AFP utiliza también los dos primeros criterios y adiciona la importancia del tema en la agenda pública, mientras que EFE considera el impacto de la afirmación o contenido que se “viraliza”. El análisis posterior implica la localización de las fuentes primarias, las imágenes, el contraste de los datos y entrevistas para obtener declaraciones.

AFP demuestra transparencia en su práctica de verificación al declarar la búsqueda inversa de imágenes, el empleo de motores de búsqueda, y algunos indicadores y herramientas para el análisis de fotos y videos, así como la presencia física en el lugar de los hechos.

En el tratamiento informativo por parte de las agencias de noticias estudiadas se utilizan elementos que reafirman o refutan el hecho, o declaran abiertamente que no se logró establecer totalmente su grado de veracidad/falsedad. Los textos periodísticos verificados se conforman mediante datos, procedimientos, comparaciones, antecedentes y declaraciones de especialistas (médicos, profesores, investigadores) y otras fuentes fidedignas (instituciones oficiales, comunicaciones científicas, traductores) que sustentan la validación. De esta forma, el proceso de *fact-checking* les revela zonas geográficas y sitios digitales en los que es frecuente la propagación de desórdenes informativos (13).

Esta estrategia de validación genera una pedagogía de verificación de contenidos —útil tanto para las personas que producen información como para las que la consumen— y alerta sobre la necesidad de tener audiencias más activas y selectivas, y emisores más éticos. La función educativa de las agencias de noticias y el periodismo se materializa también en la estrategia de alfabetización mediática de Reuters, que ha desarrollado un curso virtual dirigido a frenar los efectos de la desinformación (14). EFE, por su parte, ha creado la página temática electrónica EFE Salud, con contenidos informativos especializados y de servicio que ofrecen consejos y recomendaciones para elevar la calidad de vida.

En síntesis, estas experiencias develan no solo la utilidad sino también la necesidad de que los medios de comunicación recurran al uso del análisis de los datos, el periodismo de servicio, la anticipación y prevención de información falsa, la colaboración, la narrativa a través de diferentes medios de comunicación y la alfabetización mediática tanto de los consumidores de la información como de los que la producen.

## PROPUESTA PARA EL MANEJO DE LA INFODEMIA EN MEDIOS DIGITALES

Las recomendaciones que se presentan aquí para la gestión informativa durante la infodemia aúnan presupuestos de las ciencias de la comunicación y la epidemiología. Concebidas para enfrentar éticamente el grave problema de desinformación y mala información que enfrenta la humanidad, pueden flexibilizarse según las particularidades de los medios que las aplican.

### Etapa 1: Recogida de información

Debe enfocarse en el monitoreo e identificación de temas sospechosos, focos de atención o *trending topics,* y tendencias de búsquedas en Internet; para ello se pueden aplicar técnicas de infovigilancia e infodemiología que permitan alertar sobre posibles contenidos falsos que se “viralizan” en las redes. En particular, se deben emprender las acciones siguientes:

- Establecer vías de contacto (por ejemplo, mediante correo electrónico) para que los usuarios envíen al medio de comunicación propuestas de verificación de hechos/contenidos dudosos.
- Utilizar sistemáticamente herramientas como *Google Trends* para el desmontaje y análisis de las tendencias de búsquedas que resulten alarmantes, en especial en tiempos de contingencias epidemiológicas.
- Conformar equipos para monitorear las principales redes sociales a fin de identificar los sitios digitales y las regiones geográficas más sensibles a la circulación de contenidos falsos.

### Etapa 2: Selección de la información periodística

Se debe continuar el análisis de contenidos y tendencias, especialmente en tiempos de epidemia e infodemia, a partir de fuentes confiables —como revistas científicas y académicas, y organizaciones científicas reconocidas— y el empleo de habilidades y herramientas que posibiliten verificar los datos, confrontar las fuentes, desmontar imágenes y videos, entrevistar a especialistas y consultar sitios en Internet de organizaciones libres de bulos; se incluye aquí el tratamiento periodístico del tema. Entre las acciones de esta segunda etapa se encuentran:

- Aunar fuentes verídicas y clasificarlas para contrarrestar la desinformación en el menor tiempo posible; páginas como *Mythbusters* (https://www.who.int/emergencies/diseases/novel-coronavirus-2019/advice-for-public/myth-busters), de la OMS, ofrecen información verificada sobre la COVID-19.
- Dar la mayor prioridad al tratamiento periodístico según los valores de la noticia y criterios fundamentales, como la trascendencia, la “viralidad”, y los niveles de información y de opinión; encauzar el producto informativo hacia secciones generales o especializadas.
- Promover el periodismo de precisión, de datos, prospectivo, de verificación y de servicio (incluida su variante de prevención) para diversificar las propuestas del medio. Es importante crear secciones lideradas por periodistas de experiencia especializados en temas de salud. Considerar el empleo de formatos o géneros informativos propios de las agencias —como noticias breves (*flashes*), boletines e informes urgentes, entre otros—, así como de infografías, para difundir —y en su caso desmentir— contenidos de alto impacto sanitario, con mensajes breves e inmediatos.

### Etapa 3: Presentación de la información

Conlleva acciones que contribuyen a la mitigación de daños, mediante una comunicación que logre anticiparse, prevenir o contrarrestar los efectos negativos que pueda provocar la infodemia. Para ello es preciso:

- Presentar una evaluación sobre los contenidos objeto de verificación, sustentada en principios éticos que contribuyan a elevar el prestigio del medio y que, a su vez, desarticulen el contenido falseado.
- Crear mecanismos de difusión que no solo se centren en el soporte tradicional del medio, sino que se expandan a redes sociales y otros canales para ampliar el consumo y el seguimiento por parte de los receptores.
- Establecer vías de retroalimentación e interactividad con las audiencias mediante diferentes canales, como el correo electrónico del medio, *chats*, redes sociales, comentarios de publicaciones y consultorios (intercambios directos entre médicos y periodistas especializados con el público para atender sus interrogantes y preocupaciones a través de una sección específica).
- Participar activamente en la alfabetización mediática, ya que los medios de comunicación son también responsables de educar a las audiencias acerca del consumo informativo; en este sentido, se deben fomentar cursos en línea, tutoriales y mecanismos de transparencia informativa, con un lenguaje asequible.

Se espera que las acciones propuestas, estructuradas en etapas, sirvan de guía a las agencias de noticias y a los medios digitales en general en la sistematización de sus respuestas a las infodemias, siempre en consonancia con las fases del proceso de producción noticiosa y las de enfrentamiento a epidemias.

## CONCLUSIONES

El periodismo se debe convertir en un actor clave en el enfrentamiento a la sobreabundancia de información inexacta, engañosa y falsa sobre la salud, en particular la pandemia de COVID-19. Las experiencias positivas observadas en las agencias de noticias analizadas —ya sea mediante *fact-checking*, secciones dedicadas al tema de la salud y colaboraciones, así como con la educación en la validación de contenidos— han servido para proponer esta guía para el manejo informativo durante emergencias sanitarias e infodemias por parte de medios de comunicación digitales.

En la gestión informativa de la infodemia en medios digitales se deben interrelacionar oportuna y paralelamente las etapas del proceso de producción periodística con las del enfrentamiento a las epidemias, desde estrictos fundamentos éticos, para viabilizar la producción y el acceso a contenidos veraces.

## Declaración.

Las opiniones expresadas en este artículo son responsabilidad de los autores y no reflejan necesariamente los criterios ni la política de la *Revista Panamericana de Salud Pública / Pan American Journal of Public Health* y/o de la Organización Panamericana de la Salud.
